# Supplementary material for: Assessing the Healthy Food Partnership’s Proposed Nutrient Reformulation Targets for Foods and Beverages in Australia
Source: Nutrients. 2020 May 8;12(5):1346. doi: 10.3390/nu12051346 (PMC7285116; doi:10.3390/nu12051346)
Supplement: Supplementary file 1 [file nutrients-12-01346-s001.pdf]

Supplementary Table S1. FoodSwitch data compared to UK sodium targets

	FoodSwitch			Potential targets				
**UK category**	**Number of products**	**Mean (SD; mg/100g)**	**Median (range; mg/100g)**	**FoodSwitch 33.3 percentile (mg/100g)**	**UK average target (mg/100g)**	**UK maximum target (mg/100g)**	**Products meeting UK maximum target (%)**	**Already targeted**
Bacon	124	1110.3 (346.5)	1050 (430-2900)	997.0	1150	-	-	Y
Ham and other cured meats	144	1097.7 (382.4)	1050 (1.7-4320)	995.0	650	-	-	Partial
Uncooked sausages	186	681.6 (219.4)	649 (79-1840)	581.0	450	550	26.3%	Y
Delicatessen meat pies	195	422.6 (121.9)	405 (144-930)	365.0	390	450	66.7%	Y
Cornish meat pasties	1	562 (.)	562 (562-562)	562.0	360	400	0.0%	N
Other meat-based pastry	28	453.1 (158)	410 (220-951)	399.0	270	300	14.3%	N
Whole muscle meat	535	421.4 (282.6)	379 (30-1570)	272.0	-	270	32.9%	N
Reformed whole muscle meat	402	583.2 (259.9)	558 (3.2-1600)	449.0	-	360	19.9%	Partial
Comminuted or chopped reformed meat	63	517.2 (222.6)	496 (1-1100)	421.0	-	540	65.1%	N
Burgers and grill steaks	123	461.7 (166.9)	434 (55-1050)	386.0	300	350	23.6%	N
Fresh chilled frankfurters	30	1065.7 (233.2)	1120 (546-1400)	995.0	600	750	10.0%	Y
Bread and rolls	817	439.1 (151.6)	400 (3-1200)	390.0	360	450	66.1%	Y
Bread and rolls with additions	82	507.8 (122.1)	529.5 (210-824)	476.0	400	450	29.3%	N
Morning goods – yeast raised	143	318.8 (113.5)	310 (1.7-674)	270.0	290	350	63.6%	Partial
Morning goods – powder raised	54	436.4 (164.6)	400 (90-794)	335.0	450	500	70.4%	Partial
Breakfast cereals	941	125.3 (221.9)	44 (0-5000)	15.0	235	400	94.0%	Partial
Cheddar and other similar hard pressed cheeses	474	679.5 (233.6)	651 (1-2600)	635.0	700	800	91.6%	Partial
Fresh soft white cheeses	249	505.7 (398.7)	358 (1-1800)	300.0	200	270	26.9%	N
Cottage cheese	21	219.5 (141)	300 (32-419)	98.0	200	210	42.9%	N
Mozzarella	36	506.3 (211)	505 (130-1526.3)	417.0	540	-	-	N
Blue cheese	50	928.6 (365.1)	800 (3.6-1529)	710.0	800	-	-	N
Other processed cheese	73	1165.6 (403)	1240 (400-1950)	900.0	680	800	30.1%	Y
Salted butters and buttery spreads	83	507.2 (209.5)	481 (9-1139)	380.0	590	670	74.7%	N
Lightly salted butter	27	15 (19.3)	10 (0-100)	6.0	450	-	-	N
Margarines/other spreads	136	343.8 (239.5)	350 (0-1300)	340.0	425	550	88.2%	N

Baked beans in tomato sauce without accompaniments	79	383.5 (480.3)	354 (9-4500)	280.0	-	225	10.1%	N
Ready meals and meal centres	1068	306.7 (149.9)	280 (0-2055)	241.0	250	380	78.1%	Partial
Soups	543	280.2 (84.2)	278 (3-887)	260.0	210	250	26.5%	Y
Pizzas	159	544.7 (175.6)	547 (115-1230)	470.0	400	500	41.5%	Y
Standard potato crisps	296	570.7 (189.7)	540 (7-1460)	499.0	525	580	62.2%	Y
Extruded and sheeted snacks	674	612.5 (374.3)	564 (0.7-3100)	430.0	680	800	78.2%	Partial
Salt and vinegar products	44	957.6 (313.8)	859.5 (427-1950)	760.0	750	1000	63.6%	Y
Cakes	423	274.2 (151.6)	252 (0-1170)	193.0	170	280	56.7%	Partial
Pastries	77	389.9 (200.3)	334 (25-1200)	290.0	140	180	6.5%	N
Sweet pies and other shortcrust or choux pastry based desserts	124	204.6 (92)	209 (21-523)	150.0	100	130	23.4%	N
Sandwiches with/without high salt fillings	79	461.7 (156.2)	443 (120-950)	386.0	360/270	600/350	79.7%	N
Tomato ketchup	73	709.1 (327.4)	765 (0-1540)	583.0	-	680	41.1%	N
Brown sauce	75	781.5 (272.6)	770 (0-1360)	670.0	-	480	14.7%	N
Salad cream	83	762.4 (251.9)	804 (66-1210)	715.0	-	630	20.5%	N
Mayonnaise (not reduced/reduced fat/calorie)	152	597 (222.8)	573 (53-1500)	500.0	-	500/680	33.6%	N
Salad dressing	108	499.7 (527.7)	196.5 (0-1861)	20.0	-	600	56.5%	N
Cooking and pasta sauce	629	542.6 (756.6)	380 (0-8460)	338.0	300	370	46.9%	Y
Pesto and other thick sauces	247	869 (776.4)	650 (100-4940)	429.0	550	650	48.8%	Partial
Thick pastes	195	2023.1 (1861.9)	1659 (2.1-11000)	1050.0	1300	1500	46.2%	N
Sweet biscuits	1362	213.1 (138.8)	200 (0-850)	140.0	220	380	88.5%	N
Savoury biscuits	780	632.8 (327.3)	615.5 (0-2500)	490.0	520	700	61.3%	Partial
Pasta and noodles, plain and flavoured	333	741.7 (786.7)	390 (5-3110)	295.0	200	350	45.6%	N
Rice (unflavoured), as consumed	232	19.2 (58.5)	5 (0-560)	5.0	-	70	94.4%	N
Flavoured rice, as consumed	100	287.3 (200.4)	250 (0-1046)	205.0	180	230	40.0%	N
Other cereals	100	435.6 (237.7)	419.5 (0-1090)	360.0	220	250	20.0%	N
Dessert mixes, as consumed	41	160.3 (161.7)	130 (0-725)	58.0	-	180	65.9%	N
Cheesecake	58	172 (71.5)	165.85 (46-503)	136.0	110	140	37.9%	Y
Sponge-based processed puddings	51	267.4 (95)	272 (70-460)	223.0	170	250	47.1%	Y
All other processed puddings	192	172.3 (110)	150 (0-570)	104.0	70	110	33.9%	Partial
Quiches	42	451.7 (110.7)	440 (112-717)	410.0	220	270	4.8%	Y
Canned tuna	144	346.7 (119.1)	341 (120-654)	295.0	360	-	-	N
Canned salmon	72	394.1 (173.1)	365 (62-1020)	358.0	320	-	-	N
Other canned fish	382	443.4 (249.5)	400 (61-2700)	354.0	340	600	91.1%	N

Canned and bottled vegetables	461	200.7 (222.4)	200 (0-2280)	100.0	-	50	28.9%	N
Plain meat alternatives	64	223.8 (457.3)	44 (1-3447)	12.0	-	250	65.6%	N
Meat-free products	124	517.9 (550.5)	455 (10-6000)	370.0	360	500	62.9%	N
Meat-free bacon	4	804.5 (109.1)	791 (712-924)	712.0	-	750	50.0%	N
Other processed potato products	97	213.6 (148.8)	225 (0.4-709)	76.0	185	275	62.9%	N
Dried beverages	337	113.1 (579.4)	36 (0-8300)	17.0	-	60	74.2%	N
Stocks	153	300.6 (173.4)	292 (14-1100)	242.0	300	380	72.9%	Y
Gravy	94	491.4 (118.4)	514 (230-720)	420.0	380	450	40.0%	Y

Supplementary Table S2. FoodSwitch data compared to UK sugar targets

UK category	FoodSwitch				Potential targets			Already targeted by HFP
	**Number of products**	**Mean (SD; g/100g)**	**Median (range; g/100g)**	**FoodSwitch 33.3 percentile (g/100g)**	**AU 20% reduction in mean (g/100g)**	**UK target**	**UK 20% reduction guideline**	
Breakfast cereals	941	15 (9.1)	15.7 (0-44.3)	10.6	12.0	20% reduction	12.3	Partial (not hot cereals)
Yoghurts	703	11.9 (4.2)	12.1 (0-34.8)	10	9.5	20% reduction	11.0	Y
Biscuits	1353	30.6 (11)	30.6 (0-77)	25.6	24.5	20% reduction	26.2	Partial (muesli bars only)
Cakes	443	32.1 (10.2)	31.6 (0.2-61.8)	28.3	25.7	20% reduction	27.9	N
Morning goods	197	14.7 (9.4)	14.4 (0.1-52.4)	9.9	11.8	20% reduction	10.0	N
Puddings (i.e. desserts)	280	33 (11.5)	34.3 (2.5-91.7)	27.2	26.4	20% reduction	15.1	N
Ice-cream, lollies, and sorbets	712	25.1 (7.9)	23.8 (0-56.2)	21.8	20.1	20% reduction	18.6	N
Chocolate confectionary	1593	46.8 (14)	49.9 (0-99)	44.7	37.4	20% reduction	43.7	N
Sweet confectionary	781	56.4 (22.2)	58.1 (0-100)	49.2	45.1	20% reduction	48.4	N
Chocolate spread	46	47.1 (14.5)	51.7 (9.6-67.1)	48.8	37.7	20% reduction	43.8	N
Peanut butter	78	8 (4.2)	6.5 (4.1-25.2)	5.5	6.4	20% reduction	3.9	N
Dessert toppings/sauces	210	57.9 (22.6)	55.7 (0-99.3)	45.9	46.3	20% reduction	38.7	N
Fruit spreads	31	57 (6.2)	57.5 (46.2-68.8)	53.4	45.6	20% reduction	34.6	N
